# Five-year results of a randomized clinical trial comparing a polypropylene mesh with a poliglecaprone and polypropylene composite mesh for inguinal hernioplasty

**DOI:** 10.1007/s10029-012-0916-3

**Published:** 2012-05-10

**Authors:** K. Bury, M. Śmietański, The Polish Hernia Study Group

**Affiliations:** 1 Department of Vascular and Cardiac Surgery, Medical University of Gdańsk, ul. Dębinki 7, 80-211 Gdańsk, Poland; 2 Department of General and Vascular Surgery, Ceynowa Hospital, ul Jagalskiego 10, 84-200 Wejherowo, Poland

**Keywords:** Inguinal hernia, Lichtenstein, Lightweight mesh, Recurrence, Pain

## Abstract

**Background:**

The aim of this study was to assess whether partially absorbable monofilament mesh could influence postoperative pain and recurrence after Lichtenstein hernioplasty over the long term.

**Methods:**

Patients were randomized into two groups that were treated with lightweight (LW) or heavyweight (HW) mesh in 15 centers in Poland. A modified suture technique was used in the lightweight mesh group. Clinical examination was performed. A pain questionnaire was completed five years after the surgery.

**Results:**

Of the 392 patients who underwent surgery, 161 (90.81 %) of 177 in the HW group and 195 (90.69 %) of 215 in the LW group were examined according to protocol, a median of 62 (range 57–66) months after hernia repair. There was no difference in the recurrence rate (1.9 % LW vs. 0.6 % HW; *P* = 0.493). There were 24 deaths in the follow-up period, but these had no connection to the surgery. The patients treated with LW mesh reported less pain in the early postoperative period. After five years of follow-up, the intensity and the presence of pain did not differ between groups (5 patients in the LW and 4 patients in the HW group). Average pain, (VAS score), was also similar in the LW and HW group (2.25 vs. 2.4) at the fifth year postoperatively.

**Conclusion:**

The use of partially absorbable mesh reduced postoperative pain during the short-term postoperative period. No difference in pain or recurrence rate was observed at 60 months.

## Introduction

Tension-free mesh repair currently represents the gold standard in inguinal hernia surgery [1]. To date, meshes of many different shapes, varied chemical structure, and differing designs have been introduced into medical practice. The Lichtenstein method is still the most popular. Although the use of prosthetic materials reduced the rate of recurrence [1], it may be associated with chronic pain [2, 3].

Meshes built of dense woven propylene, the substance most commonly used in the production of prosthetic materials in hernia surgery, are characterized by low biocompatibility [3]. This feature induces a potent immunologic response from the surrounding tissue, which may lead to the development of chronic pain, which was assessed in many trials to be present in more than 20 % of patients [4]. Efforts to address this problem over the last decade have led to the technological development of a new generation of lightweight meshes. Theoretically, these meshes should decrease the inflammatory response and reduce the rates of chronic pain. Recently published trials have proven that the use of lightweight meshes reduces the rate of chronic pain rate over the short term as compared to heavyweight polypropylene meshes [5–8]. Nonetheless, an increased rate of recurrence was demonstrated in one trial, suggesting the need for further characterization of this material.

The aim of this study was to assess whether partially absorbable monofilament mesh could influence postoperative pain and the recurrence of pain after tension-free hernioplasty (Lichtenstein method). Results obtained during the first month demonstrated a decreased rate of pain and no increase in the risk of recurrence. However, longer observation will be required to adequately assess the rate of recurrence.

## Patients and methods

Patients aged between 20 and 75 years diagnosed with primary, unilateral inguinal hernia were eligible to participate in the study. All the exclusion criteria were published previously [8]. Patients were recruited and then operated upon in 15 selected hospitals in Poland. All patients were informed about the study protocol and provided their informed consent. The study protocol was approved by the Ethical Committee of the Medical University of Gdansk, which represented all of the participating hospitals. The randomization process was performed just before the surgery, with the use of a Wichmann–Hill pseudorandom number generator (modified by McLeod).

All patients were operated upon by experienced surgeons using the standard Lichtenstein procedure. Investigators could not participate in the study unless each had performed a minimum of 200 Lichtenstein procedures. Before the start of the study, a workshop related to operative technique and its modification was conducted for all trial participants.

The heavyweight polypropylene mesh (Prolene; Ethicon, Hamburg, Germany) that was used weighed over 80 g/m^2^. The other alternative was a lightweight, braided, monofilament mesh with large pores made from poliglecaprone and polypropylene (Ultrapro; Ethicon, Hamburg, Germany). Analyzing the properties of Ultrapro mesh, we noted that after shaping the mesh, the pores of some margins seemed to be closed. Suturing and straining can unravel those pores and rip the suture. Based on these findings, and on the mesh elasticity, three suturing modifications were applied to the basic Lichtenstein technique in the lightweight group. A larger suture margin (minimum four pores of lightweight mesh) and about a two times shorter distance between the steps (maximum 1 cm) was used for running suture on the inguinal ligament. One additional suture was placed to fix the mesh near the pubic bone between the pubic tubercle and the middle line.

The specific anesthesia, prophylactic antibiotics, postoperative analgesic procedures, and drugs used have been published previously [8].

All data were collected on paper forms by a blinded surgeon during planned follow-up visits. Filled forms were sent to the steering committee secretary in Gdansk. The information therein was entered into the computer database by one of the investigators. Before closing the database, all files were checked to find any additional unreported complications or serious violation of the study protocol. The immediate and short-term postoperative results were published in a previous publication [8].

After five years had elapsed from the time of the surgery, all participating patients were contacted by telephone or in person by one of the investigators. Patients reporting groin pain or foreign body sensations, as well as those suspected of recurrence, were physically examined. If there was any doubt, USG was performed. The VAS scale (1–10) was used to assess pain.

### Statistical analysis

The hypothesis was that using lightweight mesh would result in less pain during the short- and long-term follow-up periods.

The number of patients required for statistical power in each group was calculated under the assumption that a reduction in pain incidence from 20 to 10 % at three months would be clinically significant. With this assumption, 80 % test power, and an alpha level of 0.05, it was determined that 210 individuals were needed in each group. Ultimately, 300 patients were included in each group due to the anticipation of patient losses. The details relevant to patient allocation were previously published [8]. It is to be mentioned that exclusion of the hospitals was conducted to avoid main bias often find in the RCT’s. The investigators inherent bias could potentially lead to positive false results in the composite mesh group. Due to this fact, the monitoring committee has excluded whole patients lists (in the case of serious violation of the protocol found in more than 1 % of patients CRF) from the center (both groups) to avoid also the exclusion bias (elimination of worst results in the treated group) in the study.

All the statistical calculations were performed using Statistica 7.1 PL (Polish version) software (StatSoft, Tulsa, Oklahoma, USA). The tests used were as follows: Student‘s *t* test, Shapiro–Wilk’s *W* test, Mann–Whitney‘s *U* test, ANOVA, and chi-square test (with Yate’s correction when needed and double-sided to check the alternative hypothesis that the probability of an observation is less than expected under the null hypothesis). *P* < 0.05 was considered statistically significant. Multivariate analysis was performed to find factors influencing postoperative pain.

## Results

A total of 600 men were randomized, 300 to standard treatment and 300 to treatment with the LW mesh. After monitoring visits, there were 215 patients in the LW mesh group and 177 in the HW mesh group. There were no significant differences between the groups [8].

**Fig. 1:**
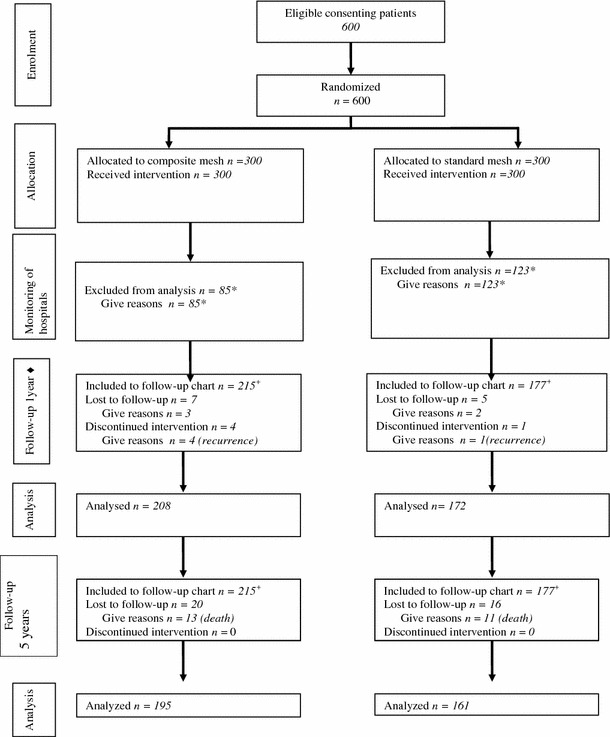
Randomized trial of composite lightweight or polypropylene mesh in Lichtenstein primary inguinal hernia repair. *plus sign* Included into final analysis after the monitoring visits in the hospitals, *asterisk* exclusion after monitoring visits in hospitals—see description in section: study flow chart; *filled diamond* published previously [8]

Of 392 patients who underwent surgery, 161 (90.81 %) of 177 in the polypropylene mesh group and 195 (90.69 %) of 215 in the poliglecaprone and polypropylene composite mesh group were examined according to protocol, a median of 62 (range 57–66) months after hernia repair. There were 24 deaths during the follow-up period, but without any connection to the surgery (Fig. 1).

The first follow-up study reported a total of five hernia recurrences, four in the lightweight group and one in the polypropylene mesh group, after 1 year [8]. The recurrence rate had not increased at the time of this clinical assessment, yielding recurrence rates of 1.86 % (2.05 % if dead patients or lost to follow-up were excluded) and 0.57 % (0.62 % if dead patients or lost to follow-up were excluded), respectively, after LW and polypropylene mesh repair (*P* = 0.493).

There were no major differences in response to the pain questionnaire. Four patients in the heavyweight mesh group and five patients in the lightweight mesh group were reporting pain. The average VAS score (with respect to pain) was 2.25 (range, 2–3) in the LW mesh group and 2.4 (range, 2–3) in the polypropylene mesh group. This difference was not statistically significant. Noninvestigating center was found as a high volume one for recurrence or pain.

In a multivariate analysis made for the whole group of patients, two factors were found as influencing the presence of pain after the operation type of the mesh used (in 3 month postoperatively [*P* = 0.046] and the presence of pain before the operation noted in 3 month [*P* = 0.0007] and 6 month [*P* = 0.0017] after operation.

## Discussion

In this multicenter study, we showed that using partially absorbable lightweight mesh as opposed to standard heavyweight polypropylene mesh did not result in an improved pain profile or a different recurrence rate at the time of 60-month follow-up.

In recent years, many studies have shown that the use of mesh decreases acute pain (up to 12 months) in comparison to tension methods. Acute pain affects up to 20 % of patients who undergo surgery with mesh [9–11]. Experimental studies showed that the extent of the foreign body reaction, with the scar tissue and inflammatory reaction it provokes, depends on the amount and physical structure of the prosthetic material that is used [4]. To solve this problem, medical companies are trying to improve the materials available and introduce new ones. Trials published by Śmietański et al. [12], O’Dwyer et al. [6], and Post et al. [5] confirmed that reducing the weight of the mesh by adding an absorbable component decreases the level of pain by half over the short term (3–6 months postoperatively). However, Bringman et al. [7] did not find significant differences in pain scores at 1 and 3 years postoperatively. Unfortunately, no metaanalysis of the trials mentioned previously has been performed. Notably, pain score can vary depending on the protocol used in a particular study. Bringman reported an incidence of pain that ranged from 1 to 24 % during the first 3–6 months. This difference disappeared after 6 months of observation, due to the low incidence of pain in both groups. Other trials have supported the use of a lightweight material [12]. Only one trial comparing weight-reduced material over the long term is currently available. This study reported the lack of any influence of the mesh on the long-term rate of pain after 60 months of observation, but the lightweight mesh used varied in design from that used in other trials [12]. Notably, Ultrapro is more elastic and macroporous in construction than Surgimesh WN (a polypropylene plate made from microfibres), which should result in the formation of a more elastic scar in the inguinal canal. Still, our findings did not confirm that the difference in weight of the mesh is responsible for the 0.43 % frequency of pain among patients treated with Ultrapro as opposed to the 2 % frequency of pain among those treated with the composite mesh [12]. Use of the lightweight material as compared to the heavyweight mesh did not provide the patient with any benefit over the long term. Notably, the frequency of pain among patients in the polypropylene group was less than 2 %, which is comparable with values reported by other groups [1].

Over recent decades, the use of prosthetic material has reduced the recurrence of hernia. The development of new materials has renewed focus on the topic. In some studies, the recurrence rate was higher among patients treated with lightweight mesh, but this trend was significant (5.6 %) in only one group [6]. O’Dwyer stated that the high rate of recurrence is a consequence of the prosthetic material’s physical features, which require modification of the fixation technique that is commonly used. The modifications applied in our trial reduced the recurrence rate to an acceptable level of 2 % during the first 5 years postoperatively, similar to the level reported by the case series that treated patients using Ultrapro mesh [13]. It is important to note that in different case series published for Ultrapro mesh the recurrence occurred mostly in the patients treated by residents in emergency settings or in patients with serious comorbidities, who were excluded in our trial, what can potentially influence the results (false positive effect of all RCT’s) [13]. The used modification must also be introduced in surgical practice before launching the mesh in a new hospital to avoid the increase in recurrence (like in O’Dwyer trial) [6]. In the presented trial, we have noted that the recurrences occurred in the first postoperative year, what corresponds with another long-term observational studies, for example, conducted by van Veen et al. (recurrence in the mesh group noted in first 2 years postoperatively only) [10]. Still it is possible that in our trial some recurrences could been missed (asymptomatic small recurrence) due to telephone examination of the patients.

We believe that presented trial adds also a new material potentially influencing the guidelines in inguinal hernia surgery [14]. Authors of the EHS guidelines have recommended the use of lightweight material considering the potential increase in recurrence rate, according to different results of available trials. The data from our study and other randomized studies might influence the conclusion of the EHS guidelines on lightweight materials.

## Conclusion

Data collected after 60 months of observation did not show any significant difference in recurrence and pain between the lightweight mesh and heavyweight mesh groups, confirming previously published conclusions about its use. When considering short-term follow-up as a primary endpoint, a lightweight material should be the first choice, despite the lack of any benefit for patients over the long term.

## Appendix

The Polish Hernia Study Group are:

Project leader: Śmietański M (Gdańsk).

Steering committee: Bigda J (Gdańsk), Łukasiewicz J (Gdańsk), Łukiański M (Gdańsk), Witkowski P (New York, USA).

Scientific advisory board: Matyja A (Kraków), Śledziński Z (Gdańsk), Śmietańska IA (consultant anesthesiology, Gdańsk), Owczuk R—(consultant statistician, Gdańsk).

Trialists: Bierca J (Warszawa), Bury K (Gdańsk), Dideńko W (Warszawa), Gąsiorowski A (Zgorzelec), Gebuza A (Wrocław), Gumela P (Gdańsk), Jędrasiak D (Warszawa), Kamiński Z (Biała Podlaska), Kontny T. (Bydgoszcz), Kniaź M (Wrocław), Kostewicz W (Warszawa), Kurzyński M (Zgorzelec), Kwiatkowski A (Warszawa), Leszczyszyn J (Wrocław), Łebski I (Wrocław), Malińska K (Środa Wlkp.), Mazur A (Kędzierzyn-Koźle), Olejarz A. (Bydgoszcz), Orłowski P (Warszawa), Paradowski T (Bydgoszcz), Paśko K (Wrocław), Perczyński W (Warszawa), Piotrowski R (Koszalin), Ryll P (Środa Wlkp.), Sachanbinski T (Opole), Sawicki M (Warszawa), Skoczylas M (Wrocław), Solecki R (Kraków), Szczepanowski A (Kędzierzyn–Koźle), Timler D (Łodź), Trojanowski P (Warszawa), Wiśniewski W (Warszawa).
